# A PEEK-Based Pedicle Screw System for One-Level Lumbar Spinal Canal Stenosis: An Appraisal at a Five-Year Follow Up

**DOI:** 10.3390/jcm14124252

**Published:** 2025-06-15

**Authors:** Andrei George Anghel, Jonas Garthmann, Baraa Alkahawagi

**Affiliations:** Spine Unit, Orthopaedic Clinic Hessisch Lichtenau, 37235 Hessisch Lichtenau, Germany; jgarthmann@lichtenau-ev.de (J.G.); balkahwagi@lichtenau-ev.de (B.A.)

**Keywords:** semi constrained screws, dynamic instrumentation, degenerative spondylolisthesis, spinal canal stenosis, peek rods

## Abstract

**Background:** This study aimed at delivering first clinical results after the use of a screw-and-PEEK rod system. Emphasis was placed on the ability of the construct to prevent adjacent segment disease at an average of 5 years follow up. **Methods:** The cohort was made up of 33 patients who received decompressive surgery in one segment and instrumentation with a screw-and-PEEK rod-based construct for stenosis of the lumbar spinal canal and a control group of 20 who received fusion surgery. **Results:** At an average of 68 months follow up there were 19 patients where the symptoms had markedly improved or completely subsided. There were also nine patients where the symptoms initially subsided only to reoccur years later and five who had a subjective non-satisfactory result. **Conclusions:** The system showed no major disadvantage when compared to similar non-fusion pedicle-based techniques, nor was it able to consequently prevent ASD. Under a clinical point of view, there was, in our opinion, no marked benefit when compared against decompressive surgery and fusion as the accepted standard.

## 1. Introduction

This article aims to further elaborate on the one-segment instrumentation of the lumbar spine with a novel PEEK-based pedicle screw system that was first developed in our clinic. An initial appraisal of the system in a larger cohort with one-, two-, and three-segment instrumentations has already found the system to be worthy of consideration for degenerative spinal canal stenosis with first-degree spondylolisthesis in one segment of the lumbar spine, as the system requires a reduced operating time in comparison to fusion techniques [1]. To further understand the advantages and disadvantages of the system in question, the patients were matched against a control group who underwent a fusion operation in our clinic.

The system was designed to avoid the development of adjacent segment pathology that is often attributed to fusion within the lumbar spine. Many other dynamic instrumentation constructs have found their way into the daily practice of surgeons around the world, but only a few have offered good outcomes in the long run.

The development of adjacent segment pathology is likely due to abnormal biomechanical stress; however, there is yet to be a definitive answer about whether it is the operation itself that generates this stress or the normal development of the spinal disease [1,2].

Greater global population growth means there is an increase in the need for spine surgery and instrumentation, which may result in an increased incidence of adjacent segment pathologies [1,3,4,5].

Other studies have analyzed the outcome of PEEK-derived instrumentation devices on the lumbar spine in a two-year follow up; however, they do not address the use of SC screws and were mostly carried out using a topping-off technique. The results are less than optimal, showing large failure rates [1,6]. Different systems based on PEEK rods have better results, but this has only been shown in studies with small sample sizes [1,7].

Among spine surgeons around the world, there is a general agreement that avoiding rigid instrumentation may provide some protection against ASP [8]. This is one of the reasons why this type of instrumentation could be used to stabilize the index segment in patients exhibiting stenosis and slight degenerative spondylolisthesis [1,9].

For patients suffering from a symptomatic spinal canal stenosis and mild olisthesis, an alternative to the well-tried fusion operation currently exists, which could be beneficial in avoiding the risk of developing relevant osteoarthritis or worsening the slippage in the index segment [10].

Patients with these symptoms could benefit the most from the construct made up of semi-constrained screws, normal Viper screws, and PEEK rods. The combination of the different components ensures adequate stability to avoid worsening the slippage while preserving some movement in the index segment, thus reducing the risk of ASP. By avoiding interbody fusion operations, patients can benefit from reduced surgical times and complications seen in those types of surgeries [1,11].

## 2. Materials and Methods

This retrospective double-arm study analyzed the outcomes of 53 patients treated in our spine unit over a year. The patients returned after surgery for ordinary clinical and radiological checkups with appointments at 3, 12, and 60 months. Clinical outcomes as well as radiological results were assessed. The patients were assigned to a surgery condition without randomization when the criteria for surgery were met. To better understand the system, the patients were compared against a control group of 20 fusion surgeries performed in the same one-year period in our clinic, and we only analyzed the one-segment instrumentations.

We obtained approval to conduct the study from the Ethics Committee of the Land Hessen.

All patients being considered for surgery had a history of intractable pain due to lumbar stenosis where conservative therapy had proved unsuccessful over the long term. Along with neurogenic pain, the patients also exhibited leg and lower back pain. The conservative therapy consisted of pain medication and periradicular infiltration therapy, as well as physiotherapy for at least 6 months. Furthermore, the patients had a spondylolisthesis of I°, which was verified using standing X-rays of the spine. The inclusion and exclusion criteria for the construct are shown in Table 1. The indication for surgery is in line with the international recommendations [12].

## 3. Implants and Surgeries

For the dynamic instrumentation, we used a construct made up of polyaxial screws, known as semi-constrained (SC) or normal screws, which allow for a good range of motion even after tightening the rod within the screw head. The screws were joined together on the left and right sides of a polyetheretherketone (PEEK) rod with a diameter of 5.5 mm. All 33 patients benefited from the same construct over one segment [1]. In the control group of 20 patients, the instrumentation was performed using the normal Viper screws of the Expedium System and a Tezo T Cage from Ullrich. The TLIF technique was used for instrumentation.

**Figure 1:**
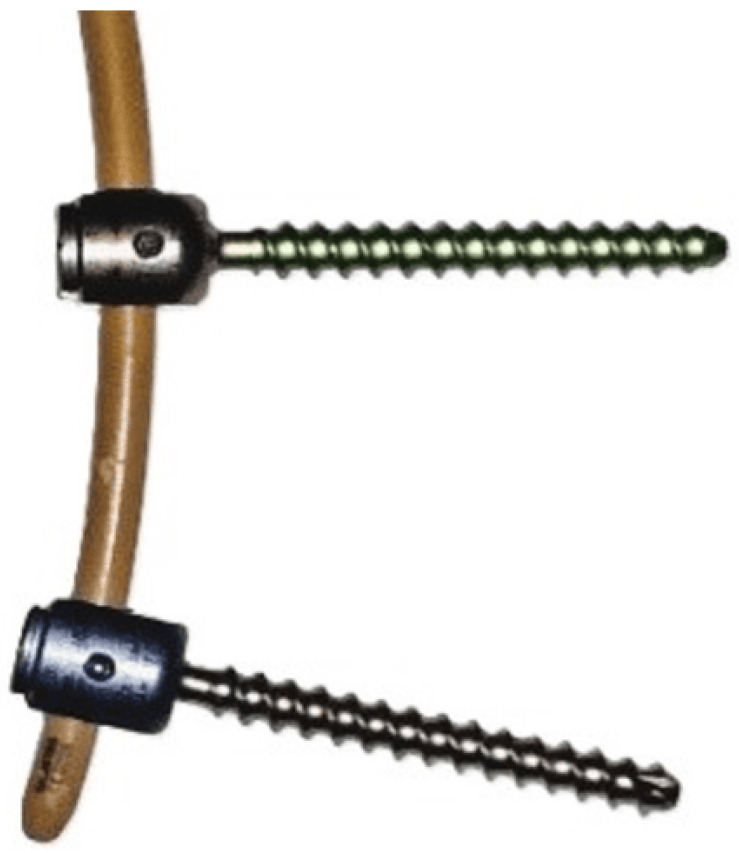
SC and normal Viper screws as well as a PEEK rod. Images are published under a Creative Commons license [1].

In the dynamic construct, the placement of the screws was always the same: in the cranial vertebrae, a semi-constrained (SC) screw was placed, and in the caudal vertebrae, a normal Viper screw was placed, as shown in Figure 1.

The surgeons performing the surgeries all have at least five years of experience and have performed the same operating procedure for their respective patients [1].

**Figure 2:**
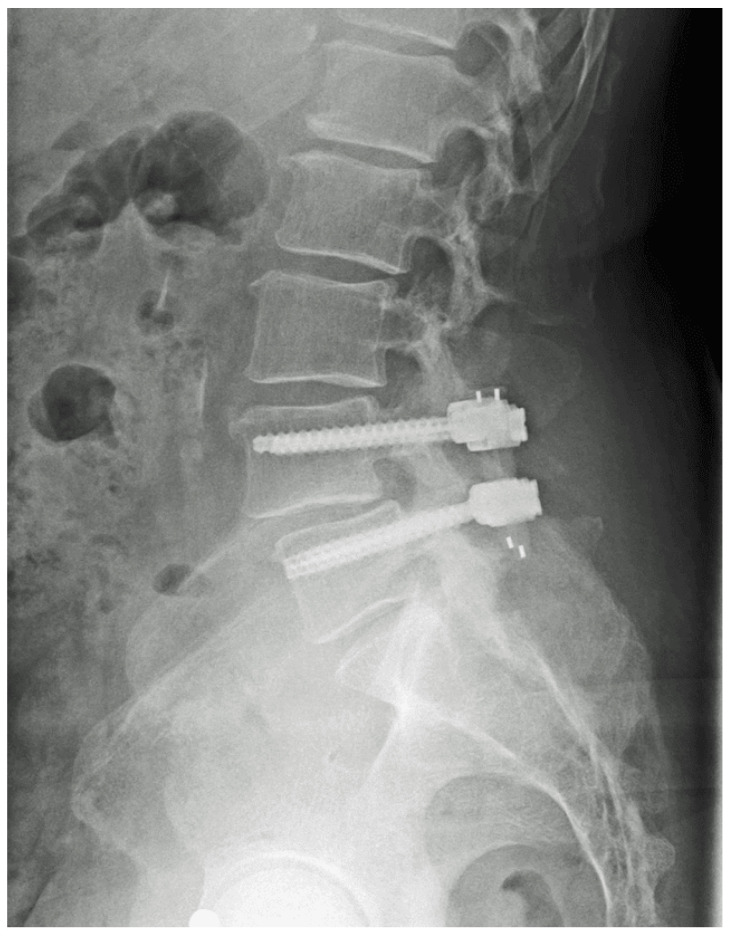
Postoperative X-ray after decompression and instrumentation at L4/5. Images are published under a Creative Commons license [1].

The first step of the surgery required the placement of the screws, with the SC screw being placed cranially and the normal Viper screw being placed caudally. After this, central decompression was performed, the results of which can be seen in Figure 2.

As a technical detail, only a moderate reduction was induced in the facet joints to try to minimize the risk of an operation-induced instability. We used intraoperative fluoroscopy to control the positioning of the screws. We positioned the screws in a manner that would not interfere with the zygapophyseal joint, ensuring that it was parallel to the upper end plate of the corresponding segment [1,11]. A torque wrench was used to tighten the rod within the screw heads.

We avoided any application of distractive or compressive force to the index segment, and no attempts to reduce spondylolisthesis were undertaken [1,13].

In preparation for the surgery, the patients underwent a clinical exam, and standing X-rays and MRIs of the lumbar spine were carried out.

## 4. Data Collection and Outcomes

Patient-reported outcomes were set as the primary end point, and postoperative complications were set as the secondary end point. Standing X-rays, including functional X-rays, were obtained after surgery [1]. We only performed standing X-rays for the control group.

## 5. Statistical Analysis

To analyze a potential correlation between gender, the area of the spinal canal, previous surgeries, and outcomes, we performed Chi-squared tests with a level of significance set at 0.05; however, we were unable to demonstrate a correlation between the abovementioned parameters at this level. All statistical analyses were conducted using IBM SPSS 25 [1].

## 6. Results

Fifty-three patients between 39 and 82 years, with an average age of 68 years, took part in our study; specifically, for women, the mean age was 65, and for men, it was 69. In our cohort, the patients were around 5 years older compared to the age of those in other studies in the literature [14]. Of the total number of participants, 32 (60%) were women, and the remaining 21 (40%) patients were men; therefore, our study has a slightly better representation of women.

In total, 51 patients exhibited a high-grade reduction in the area of the spinal canal and were appropriately classified as level D according to Schizas. Among them, 20 (39%) were men and 31 (61%) were women. The other two patients exhibited a C-type stenosis.

All patients presented with spondylolisthesis in standing X-rays of the spine. In our observations, the level where the spondylolisthesis was most often located was in segment L4/5, which was the case for 24 (72%) patients. The rest of the cohort exhibited spondylolisthesis at segments L2/3 and L3/4. In the control group, 13 (65%) exhibited spondylolisthesis at L4/5 and 7 (35%) showed it at L3/4.

Out of the total number of patients, eight have previously had surgery, with two of them being in the index segment. In the control group, four patients previously had surgeries, all in the index segment.

The average surgical time was 136 min, and in the control group, it was 167 min.

Within the observation period, the patients showed six complications when using the dynamic construct, making for 18% of the patients. There were two patients with wound infections (6%), three (9%) who developed a loosening of the implants, and one (3%) with a screw breakage. The recorded complications are acceptable and in line with those in the existing literature [15,16]. In the control group, we recorded no implant failure, but one patient (5%) had a wound infection.

Within our retrospective study, we were able to follow the subjects over a mean of 68 months, with a maximum of 96 and a minimum of 40.

Within our dynamic group, four patients received another operation in the index segment with conversion to fusion for screw loosening or breakage, and one patient required a foraminotomy in the index segment and three surgeries in other segments of their lumbar spine. The results are comparable with those of the readily available literature on this subject [16,17]. In the control group, there were no further required surgeries for the period of observation.

**Figure 3:**
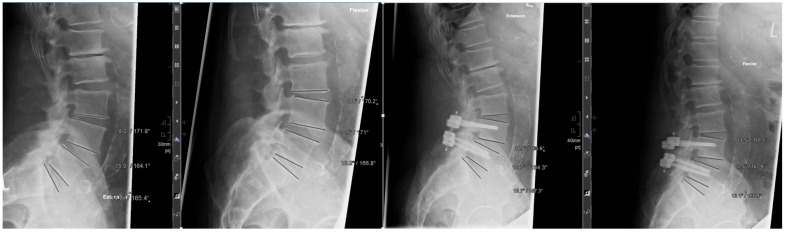
Extension/flexion X-rays with measurements of the segmental angle.

To verify the dynamic nature of the construct, we performed functional X-rays before and after the surgery with documentation of the index level, as well as the levels above and below. This can be seen in Figure 3.

**Figure 4:**
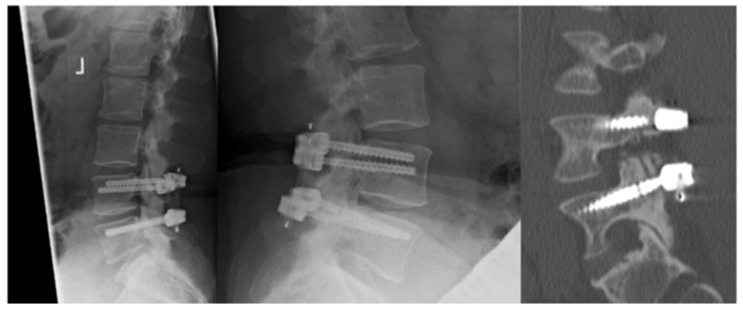
Radiological exam after surgery (**left**) and after 47 months (**middle**); CT scan (**right**).

We also carried out further investigations with CT scans when a breakage of the screws and/or of the rod was suspected in the plain X-ray. The same algorithm was used for a suspected loosening of the implants. We recorded three patients with loose screws and a case of implant breakage as seen in Figure 4.

The results in the dynamic construct indicated a rather high rate of ASP, which was found in 22 (66%) patients. Comparing these data to those provided by the existing literature is ineffective, as the incidence of ASP can be between 8 and 100% over a period of 36 to 396 months [18].

In our cohort of 33 patients, 19 (57%) had a Weiner score of 0, 8 (24%) had a score of 1, and 6 (18%) had a score of 2. At the mean follow up, 21 had exhibited a worsening; of those, 17 (51%) had a score of 0 to 1, and 4 (12%) had a score of 1 to 2. One patient had a worsening of the olisthesis from Meyerding I to II.

ASP was considered when a difference occurred in the Weiner score between surgery and the follow-up visits [19]. The same goes for the worsening of in-segment spondylolisthesis; here, it was the difference in the Meyerding classification that prompted the designation of ASP [20,21]. 

Of the total number of patients with a dynamic construct, 19 (57%) had no pain at all after surgery, 9 (27%) described an improvement in their symptoms, and the rest reported no significant improvement after surgery. In the control group, 13 (65%) were pain-free, 4 (20%) described an improvement, and 3 had no marked benefit from the surgery.

## 7. Discussion

Within the field of motion-preserving implants for the lumbar spine, many devices have been introduced to the market, but they often lack sufficient data and, more importantly, long-term information regarding the outcome of the patients.

Implants such as the Graf ligament, Dynesis, or Cosmic developed throughout the years are widely used by the community of spine surgeons, although a consensus on the real advantages of these implants is yet to be achieved [22,23,24,25].

In essence, the goals are the same: normalizing the motion pattern within the spine segment to reduce overstress and axial pain or to accompany decompression, for example, and using the system in question to avoid a worsening of the slippage. This was best observed in the lack of modification of the Meyerding score, where only one patient exhibited a worsening over the mean follow up.

Just as Graf and later Richards and Kanayama showed in their respective studies [22,23,25], it is of upmost importance to respect the limitations of the constructs when indicating the use of motion-preserving implants. If strict adherence to the use indications of non-fusion implants is given, then one can expect good results in the long term, a fact also shown in the study of Hoppe [21]. Compared to other pedicle-based constructs like Dynesis, our construct achieves similar results, with adequate reduction of neurogenic pain and a certain retention of segmental movement [22].

Although no standardized questionnaires were readily available, we were still able to observe and document the clinical outcomes of the patients during their respective follow-up visits [1].

Data provided by the patients regarding their clinical outcome match what is expected after decompression when the main symptoms are mostly neurogenic and there is no marked lower back pain. The main symptoms here were claudicatio spinalis or radicular pain. This is in accordance with other published studies where between 68% and 82% of the treated patients benefited from the surgery [1,14,16,21].

Patients of the control group showed a better reduction in lower back pain as well as in neurogenic symptoms [26].

Clinical complications did not exceed those to be expected from pedicle instrumentation in the lumbar spine.

From the radiological findings, there was a high rate of incidence for screw loosening. This shows just how much strain the construct must endure, amounting to 1 to 2 million cycles a year for a normal person. These results are in line with other studies that test pedicle screw constructs using PEEK rods [27].

On the other hand, the result partly speaks for the resilience of the PEEK rods, being able to withstand repetitive loading and unloading phases over long periods of time. Although a certain degree of movement is allowed by the rods and the SC screws, this seems to not always correspond to the individual needs of the patient, leading at some point to either a failure of the implant or a screw loosening.

This realization is what defines one of the complications of the construct, namely the stiffness of the construct, which in time may lead to a failure of the components.

The complications did not exceed those seen in similar constructs such as the one of Oikonomidis [6]. He also reported a high rate of ASD and implant failure, most probably because no off-the-shelf implant can address the complex kinematics of a specific patient and is due to fail at some point in time. Wound infections did not differ greatly from those in the literature, being observed in two (6%) of the cohort.

Some of the limitations of the study are the lack of randomization of patients and the lack of standardized questionnaires to assess the quality of life as well as the reduced cohort size. Valid extrapolation of the data thus acquired to a meaningful population size is not possible. Larger randomized studies are required to further elaborate on this subject.

## 8. Conclusions

Based on our previous data, we were able to further elaborate on one-segment instrumentation with a new device based on Viper and SC screws as well as PEEK rods and compare it to spinal fusion.

Overall, there is a non-inferiority result regarding our cohort at a five-year follow up. The observed results are similar to those provided by the literature, in terms of both clinical and radiological aspects. In our cohort, four (12%) patients developed screw loosening or breakage, and almost two thirds of them developed ASP; in contrast, there was no implant failure within the control group.

The rates of complications are similar to those typically expected in this type of surgery.

Regarding clinical outcomes, the construct is advantageous in reducing the operation time needed to perform a fusion, for example; thus, it is a good alternative, particularly in patients needing time-sensitive operations.

## Figures and Tables

**Table 1:** Inclusion and exclusion criteria.

Inclusion Criteria	Exclusion Criteria
Stenosis of the lumbar spinal canal in 1 segment Spondylolisthesis I°	Multisegmental stenosis Scoliosis of 25° or more Spondylolisthesis of II° or more

## Data Availability

The original contributions presented in the study are included in the article, further inquiries can be directed to the corresponding authors.

## Abbreviations

ASD: adjacent segment disease; ASP: adjacent segment pathology; COMI: core outcome measure index; FU: follow up; LSS: lumbar spinal stenosis; MRI: magnetic resonance imaging; PEEK: polyetheretherketone; ROM: range of motion; SC screws: semi-constrained screws.
